# Bovine fetal mesenchymal stem cells exert antiproliferative effect against mastitis causing pathogen *Staphylococcus aureus*

**DOI:** 10.1186/s13567-019-0643-1

**Published:** 2019-04-11

**Authors:** Berly Cahuascanco, Javiera Bahamonde, Olger Huaman, Miguel Jervis, Jahaira Cortez, Jaime Palomino, Alejandro Escobar, Patricio Retamal, Cristian G. Torres, Oscar Alejandro Peralta

**Affiliations:** 1 Department of Animal Production Science, Faculty of Veterinary Medicine, University of Chile, 8820808 Santiago, Chile; 2 Institute of Dental Science Research, Faculty of Dentistry, University of Chile, 8380492 Santiago, Chile; 3 Department of Preventive Medicine, Faculty of Veterinary Medicine, University of Chile, 8820808 Santiago, Chile; 4 Department of Clinical Sciences, Faculty of Veterinary Medicine, University of Chile, 8820808 Santiago, Chile; 5 Department of Biomedical Sciences and Pathobiology, Virginia-Maryland Regional College of Veterinary Medicine, Virginia Tech, Blacksburg, VA 24060 USA; 6 Present Address: Institute of Pharmacology and Morphophysiology, Faculty of Veterinary Sciences, Austral University of Chile, 5110566 Valdivia, Chile

## Abstract

*Staphylococcus aureus* is the most commonly isolated pathogen from clinical bovine mastitis samples and a difficult pathogen to combat. Mesenchymal stem cells (MSC) are multipotent progenitor cells equipped with a variety of factors that inhibit bacterial growth. The aim of the present study was to evaluate the in vitro antibacterial potential against *S. aureus* of conditioned medium (CM) from MSC derived from fetal bovine bone marrow (BM-MSC) and adipose tissue (AT-MSC). BM-MSC, AT-MSC and fetal fibroblasts (FB) cultures were activated by infection with *S. aureus*. Bacterial growth was evaluated in presence of CM, concentrated CM (CCM), activated CM (ACM) and concentrated ACM (CACM) from BM-MSC, AT-MSC and FB. Gene expression of β-defensin 4A (bBD-4A), NK-lysine 1 (NK1), cathelicidin 2 (CATHL2), hepcidin (HEP) and indoleamine 2,3 dioxygenase (IDO) and protein expression of bBD-4A were determined in activated and non-activated cells. The majority of BM-MSC and AT-MSC expressed CD73, Oct4 and Nanog, and were negative for CD34. Growth of *S. aureus* decreased when it was exposed to CM from BM-MSC, AT-MSC and FB. Moreover, growth of *S. aureus* in CCM, ACM and CACM was lower compared to controls of CM from BM-MSC and AT-MSC. Activated AT-MSC increased mRNA levels of bBD4A and NK1, and protein levels of bBD4A in CM. Thus, CM from fetal bovine BM-MSC and AT-MSC has the capacity to reduce in average ~30% of *S. aureus* relative growth under in vitro conditions. The in vitro antibacterial effect of fetal bovine MSC may be mediated by bBD4A and NK1 activity.

## Introduction

Mesenchymal stem cells (MSC) are multipotent progenitor cells with ability to self-renew and differentiate into tissues of mesodermal origin including osteoblasts, adipocytes, chondrocytes and myocytes [1]. Based on the ability to adhere to plastic when plated in monolayer culture, MSC can be isolated from several tissue sources including bone marrow (BM) and adipose tissue (AT) [2]. Plastic adherence under standard culture conditions is the first criteria established by the international society for cellular therapy (ISCT) for defining human MSC [3]. Additional criteria include trilineage differentiation potential and expression of MSC surface antigens markers CD105 (endoglin), CD73 (ecto-5′-nucleotidase) and CD90 (Thy-1), and lack of expression of hematopoietic markers CD45 (protein tyrosine phosphatase, receptor type, C), CD34 (CD34 molecule) and CD14 (CD14 molecule). The trophic capacity of MSC from adult tissues represents a promising therapeutic strategy for the treatment of a wide range of pathologies in human and veterinary medicine. Thus, there is high scientific interest in better understanding the mechanism underlying these potential therapeutic effects.

Numerous studies have reported that MSC exert reparative functions through paracrine activity including immunomodulatory effects mediated by dendritic and B and T cells, and trophic functions involving antiapoptotic, proangiogenic and mitogenic factors [4]. Moreover, a large body of evidence indicates that MSC enhance bacterial clearance through the expression of antibacterial peptides (AP) including cathelicidin (CATHL2), indolamine 2,3-dioxygenase (IDO) and hepcidin (HEP) [5–7]. CATHL2 LL-37 from human MSC is a membrane pore-forming AP that has been shown to inhibit in vitro bacterial growth of *Escherichia coli*, *Pseudomona aeruginosa*, and *Staphylococcus aureus* [5]. IDO is a key mediator of immune tolerance whose activity as a catabolic enzyme can deplete tryptophan in MSC cultures, thereby hindering growth of *S. aureus*, *Staphylococcus epidermidis*, *Enterococcus faecium* and *E. coli* [6]. Menstrual fluid derived MSC exert an important antimicrobial effect against fecal bacteria mediated by HEP [7]. HEP acts as an iron regulatory hormone that may reduce iron levels and limits its availability to microorganisms [8]. These studies suggest that MSC are equipped with a variety of factors that may be useful to fight infection in a diversity of in vivo settings.

Inflammation of the mammary gland or mastitis, manly as a consequence of infectious agents, is the most prevalent and costly disease for dairy herds worldwide. It has been reported that *S. aureus* is the most commonly isolated pathogen from clinical mastitis samples and the second most commonly isolated pathogen in subclinical samples [9]. *S. aureus* can survive in neutrophils, invade into mammary epithelial cells, form micro abscesses, and promote biofilm formation [10]. Despite in vitro antimicrobial susceptibility of *S. aureus* remains relatively high, pathogen factors make *S. aureus* a difficult pathogen to combat due to the restriction of their contact with antibiotics [11]. Consequently, percentages of cure in mastitis caused by *S. aureus* using currently approved antibiotics (e.g., pirlimycin) range only between 10 and 30% [12]. Considering that therapies available for the treatment of mastitis caused by *S. aureus* remain suboptimal, culling infected cows is a commonly used strategy to reduce new intramammary infection in infected herds.

In addition to its negative economic impact, mastitis has also been declared as the main disease threating animal welfare [13]. Bovine mastitis also affects public health by altering physical and microbiological properties of milk and increasing content of residues. Currently, treatment of mastitis is based on the use of antibiotics, aiming at controlling bacterial infection of the mammary gland. Nevertheless, this approach is not always effective and the inadequate use of antibiotics may result in bacterial resistance. Penicillin resistance is probably the most well-known antibiotic resistance of *S. aureus* with levels that differ considerably between countries ranging from 20 to 30% to more than 85% [14]. In addition, antibiotic treatment does not promote the regeneration of the mammary gland tissue, which is a crucial effect for future milk production. Thus, development of new therapeutic alternatives for the effective control and treatment of bovine mastitis is important aspect for veterinary medicine. In the present study, we aimed at evaluating the in vitro antibacterial potential against *S. aureus* of conditioned medium (CM) derived from MSC isolated from fetal bovine bone marrow (BM-MSC) and adipose tissue (AT-MSC).

## Materials and methods

### Isolation and culture of bovine fetal BM-MSC, AT-MSC and fibroblasts

BM-MSC were harvested following a previously reported protocol that ensured establishment of MSC cultures that fulfill the minimal criteria for definition of MSCs [15, 16]. BM was aspirated from male bovine fetuses (*n* = 9; 7–8 months of gestation) collected at a local abattoir. The BM was drawn from femoral cavity into syringes containing high glucose (4.5 g/L) Dulbecco’s Modified Eagle Medium (DMEM, Gibco, Grand Islands, NY, USA) supplemented with 100 IU/mL penicillin, 100 μg/mL streptomycin and 2.5 µg/mL amphotericin B (Hyclone, Thermo Fisher Scientific, UT, USA). BM samples from three fetuses were pooled and washed twice with phosphate-buffered saline (PBS) and twice with DMEM. AT-MSC were harvested from fetal abdominal omentum from the same fetuses used for BM-MSC isolation. Approximately 10 g of AT were isolated under aseptic conditions and deposited in PBS supplemented with 100 IU/mL penicillin, 100 μg/mL streptomycin and 2.5 µg/mL amphotericin B (Hyclone). AT samples from three fetuses were pooled and washed twice with phosphate-buffered saline (PBS; Hyclone) and twice with DMEM. Then, AT was digested in 0.5% collagenase I (Sigma-Aldrich, St. Louis MO, USA) (1 mL/g of AT) for 45 min. Collagenase I activity was neutralized with DMEM supplemented with 10% fetal bovine serum (FBS; Gibco), 100 IU/mL penicillin, 100 μg/mL streptomycin and 2.5 µg/mL amphotericin B (expansion medium). The disrupted tissue was filtered through 40 μm pores and subsequently centrifuged at 400×*g* for 5 min. The cell pellet was washed once in DMEM, suspended in expansion medium and plated.

Fetal fibroblasts (FB) were used as negative controls and were harvested from epithelial tissue extracted from the muzzle of the same fetuses used for BM-MSC and AT-MSC isolation. Tissue from three fetuses was isolated under aseptic conditions, pooled and washed in PBS supplemented with 100 IU/mL penicillin, 100 μg/mL streptomycin and 2.5 µg/mL amphotericin B. Tissue was washed in PBS and digested in 0.5% collagenase I at 37 °C for 90 min. Collagenase I activity was neutralized with expansion medium. The disrupted tissue was filtered through 40 μm pores and subsequently centrifuged at 400 × *g* for 5 min. The cell pellet was washed once with DMEM, suspended in expansion medium and plated. BM-MSC, AT-MSC or FB were plated in expansion media and incubated at 38.5 °C in a humidified atmosphere containing 5% CO_2_. After 2 days, non-adherent cells were removed by changing the culture medium. Following the initial 2 days, the medium was changed every 2 to 3 days. After 3 to 4 passages, cells were gently harvested when 90% confluent using 0.05% trypsin in 0.02% EDTA. Cultures of both MSC lines were characterized by quantification of CD73, CD34, Oct-4 and Nanog expression. Following determination of cell viability, cells were used to initiate experiments.

### Flow cytometry analysis

Determination of cell population positive for CD73, CD34, Oct4 and Nanog was estimated using fluorescence-activated cell sorter (FACS) analysis. Approximately 1–3 × 10^6^ cells obtained from BM-MSC and AT-MSC cultures were detached after incubation in EDTA for 10–20 min at 38.5 °C and centrifuged at 400 × *g* for 5 min. Cells were then fixed with 4% paraformaldehyde in PBS for 10–15 min at 4 °C, washed with PBS and stored at 4 °C overnight. Samples were centrifuged at 1110 × *g* for 5 min, resuspended in a solution of 1% Triton X100 in PBS, incubated during 15 min at room temperature, centrifuged at 1110 × *g* for 5 min, and then resuspended in 0.1% tween in PBS for washing. Antigens were blocked using a solution of 3% bovine serum albumin (BSA) with 1.5 mg/mL of glycine during 1 h at room temperature and then were washed with PBS and centrifuged 1110 × *g* for 5 min. Cells were incubated overnight in goat polyclonal anti-CD73, anti-CD34, anti-Oct4 or anti-Nanog antibodies (Cat. #sc-14682; sc-7045; sc-8628 and sc-30331; Santa Cruz Biotechnology, Santa Cruz, CA, USA) diluted (1:200) in 3% BSA in PBS. Cells were washed thrice in 0.1% tween in PBS, centrifuged at 1110 × *g* for 5 min and incubated in a solution of rabbit anti-goat IgG conjugated with Alexa Fluor 488 (Cat. #A11078; Thermo Fisher Scientific, Rockford, IL, USA) diluted (1:1000) in 3% BSA during 1 h at room temperature. Then cells were washed thrice in 0.1% tween and then incubated with 3 µL propidium iodide for 5 min at room temperature. Cells were resuspended in IsoFlow buffer and analyzed (three replicates) using a Gallios Flow Cytometer (Beckman Coulter, Brea, CA, USA) using a 488-nm laser light. The threshold for negative events was set on the first decade of fluorescence level histogram. Negative procedural control corresponded to cells not incubated with antibodies (autofluorescence) and cells incubated only with secondary antibody. Percentage of cells positive for autofluorescence and secondary antibody were subtracted from the percentage of cells positive with primary and secondary antibodies.

### Conditioned media collection

Conditioned media was collected from BM-MSC and AT-MSC and FB derived from the same bovine fetuses. To this end, 6 × 10^3^ cells/cm^2^ at passages 3–5 were seeded in T75 flasks with culture medium until reaching 70–80% confluency. Then medium was removed, cell monolayers were rinsed twice with PBS and 10 mL of DMEM (Mediatech, Incorporated, Manassas, VA, USA) were added. Medium was collected 72 h later, centrifuged twice for 5 min at 400 × *g* and frozen at −20 °C until used for subsequent experiments. Concentrated CM (CCM; tenfold) was obtained by filtration of CM using Amicon Ultra centrifugal filters (Merck Millipore, Tullagreen, Ireland) with a membrane NMWL of 3 kDa in accordance with the instructions of the manufacturer. Equal volumes of serum-free DMEM but without cells were handled under the same conditions and served as positive controls.

### Antimicrobial assay

Strain SAU-1S of *S. aureus* was isolated from a clinical bovine mastitis case following the recommendations of the National Mastitis Council (NMC) and then characterized for antibacterial resistance and virulence factors (Table 1). *S. aureus* SAU-1S colonies were resuspended in 2 mL of Luria–Bertani (LB) agar (Life Technologies) plates and incubated at 38 °C in shaking for 24 h. The strain was then seeded on LB agar plates at 4° for up to 1 month. For each experiment, a colony was picked and resuspended on LB broth and incubated in continuous agitation at 38 °C until cultures reached the exponential growth phase. The colonies were then washed thrice in PBS (pH 7.4), resuspended (1:100) in CM collected from BM-MSC, AT-MSC and FB, seeded in LB plates and incubated at 38° for 24 h. For direct inhibition assays, BM-MSC, AT-MSC or FB were cultured in 24-well plates (2 × 10^5^ cells per well) in serum-free DMEM and infected with 300 CFUs of bacteria for 6 h at 38 °C. Activated CM (ACM) was collected and the bacterial fraction was removed by passing the CM through a 0.22-µm filter. The filtered ACM was centrifuged at 13 200 rpm for 10 min. Concentrated ACM (CACM; tenfold) was obtained by filtration of CM using Amicon Ultra centrifugal filters (Merck Millipore) with a membrane NMWL of 3 kDa in accordance with the instructions of the manufacturer.

**Table 1:** **Characterization of strain SAU-1S of** ***Staphylococcus aureus*** **used for determination of the antibacterial potential of bovine fetal BM-MSC and AT-MSC**

Strain	*SAU*-*1S*
Biochemical tests	Catalase and coagulase positive
Source	Isolated from clinical bovine mastitis case following recommendations of the National Mastitis Council (NMC)
Antimicrobial susceptibility	Ampicillin, Cefadroxil, Cephalothin, Ceftiofur, Erythromycin, Gentamicin, Methicillin, Oxacillin, Penicillin, Tetracycline
Virulence factors	Adhesion Factor A and B, elastin-binding protein, bone sialoprotein-binding protein, protein A, hemolysin and intracellular adhesion operon A and D, moderate capacity to form biofilm

In order to determine *S. aureus* growth in CM, CCM, ACM and CACM from BM-MSC, AT-MSC and FB, bacterial proliferation was evaluated by CFUs counting at 0, 1, 2 and h post-seeding. Bacterial proliferation was expressed as percentage in relation to CFUs at time 0 post-seeding using the following formula: CFUs/mL = (mean CFUs × dilution factor) × 200; proliferation rate = (Tn × 100)/T0 where dilution factor is the counting at 1:10 000 dilution; Tn is the number of CFUs/mL at n h post-inoculation, and T0 is the number of CFUs/mL at 0 h post-inoculation.

### RNA extraction and cDNA synthesis

Approximately 3 × 10^5^ of activated and non-activated BM-MSC, AT-MSC and FB were collected and immediately fixed in RLT buffer (Qiagen, Incorporated, Valencia, CA, USA) supplemented with β-mercaptoethanol (Sigma). Total RNA was extracted using GeneJET RNA purification kit (Thermo Fisher Scientific) according to the manufacturer’s instructions. Total RNA was eluted in 50 µL of RNase free water. The concentration and purity of the RNA in each sample was determined using Qubit RNA assay kit (Life Technologies, Waltham, MA, USA), and genomic DNA was removed using DNase I, RNase-free (Thermo Fisher Scientific). Samples were subjected to reverse transcription using a cDNA synthesis kit (AffinityScript; Agilent Technologies, Santa Clara, CA, USA). The reaction protocol consisted of incubation for 5 min at 25 °C, 15 min at 42 °C, 5 min at 95 °C, and hold at 4 °C using a TC1000-G gradient thermocycler (SciLogex, Rocky Hill, CT, USA).

### Quantitative-PCR

Samples were analyzed for genes bBD4A, NK1, CATHL2, HEP and IDO expression by Q-PCR. The housekeeping β-ACTIN and GAPDH were selected as housekeeping genes based on previous analyses from our laboratory [15, 16] that detected high stability during MSC culture. Realtime PCR primers were designed using PrimerExpress software (Applied Biosystems Incorporated, Foster City, CA, USA; Table 2). Equivalence of amplification efficiencies among all primer–probe sets was confirmed using serial threefold dilutions of BM-MSC and AT-MSC cDNA. Each RT-PCR reaction (10 μL) contained the following: 2X Brilliant II SYBR Green QPCR master mix (5 μL), target forward primer (200 nM), target reverse primer (200 nM), cDNA synthesis reaction (1 μL), and nuclease-free PCR-grade water to adjust final volume. The PCR amplification was carried out in Eco Real-Time PCR System (Illumina Incorporated, San Diego, CA, USA). Thermal cycling conditions were 95 °C for 10 min, followed by 40 repetitive cycles at 95 °C for 30 s, and 60 °C for 1 min. All reactions were performed in triplicate. In each experiment, amount of gene expression was recorded as CT values that corresponded to the number of cycles where the fluorescence signal can be detected above a threshold value. The CT averages for each biological replicate were calculated and transformed into relative values denominated quantity (Q) through ΔΔCT formula [17]. Then, the relative quantification in the expression of target genes for each sample was estimated as the quotient between Q value of the target gene and a normalization factor (NF), which was calculated based on the geometric mean of housekeeping gene Q values [17].

**Table 2:** **Sequence of primers used for Q-PCR analysis**

Gene	Sense antisense	Accession number
Housekeeping genes		
GAPDH	CCTTCATTGACCTTCACTACATGGTCTA TGGAAGATGGTGATGGCCTTTCCATTG	NM_001034034.2
βACTIN	CGCACCACTGGTATTGTCAT TCCAAGGCGACGTAGCAGAG	NM_173979.3
Antimicrobial peptides genes		
bBD4A	GCCAGCATGAGGCTCCATC GGCACAAGAACGGAATACAGA	NM_174775.1
NK1	CCAGCAAGAATGTCATCATCC GTCCTTAGAGATGCGATTGAGATAC	NM_001046578.1
CATHL2	GGATTGGTGGACGAAATCTG GAATGGGCTGGTGAAACAGT	NM_174826.3
HEP	GACAGACGGCACAATGGCAC TGGAGGGCAGCAGGAATAAATA	NM_001114508.2
IDO	CGAATATACTTGTCTGGTTGG GGAGAACATCAAAGCACTG	NM_001101866.2

### Elisa

The CM, CCM, CACM from activated and non-activated BM-MSC, AT-MSC and FB were collected and centrifuged at 12 000 × *g* for 3 min at 4 °C. The amounts of bBD4A secreted into the medium were measured using an ELISA kit (Cloud-Clone Corporation, Houston TX, USA) according to the manufacturer’s instructions.

### Data analysis

Values of percentages of MSC positive for CD73, CD34, Oct-4 and Nanog, *S. aureus* relative growth, bBD4A and NK1 relative expression and bBD4A concentration from three replicates were transferred to a spreadsheet and analyzed using Infostat software. Data was normalized to logarithmic scale in base 10 for normality and mean values for each replicate were compared by one-way ANOVA. Values of *S. aureus* relative growth, bBD4A and NK1 relative expression and bBD4A concentration between treatments and controls were analyzed using Tukey’s multiple comparison test (*P *< 0.05).

## Results

### *Staphylococcus aureus* relative growth is reduced in presence of CCM from BM-MSC and AT-MSC derived from bovine fetuses

**Figure 1:**
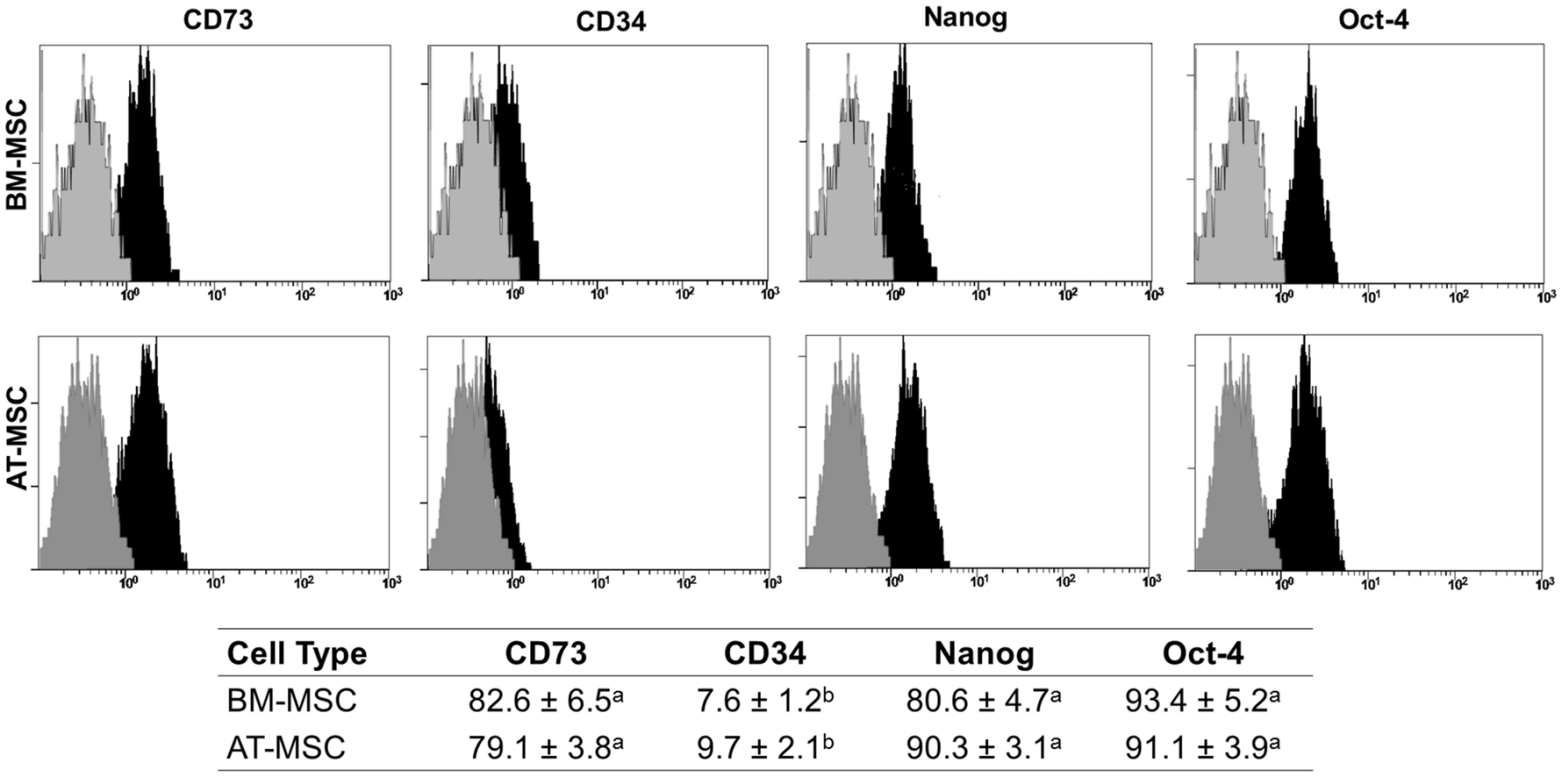
**Comparative analysis of expression of mesenchymal CD73, hematopoietic CD34, pluripotent Nanog, and Oct4 markers in bovine fetal BM-MSC and AT-MSC.** High proportion of BM-MSC and AT-MSC expressed CD73, Nanog and Oct4. BM-MSC expressed higher levels of CD34 compared to AT-MSC. (a–c) Indicate significant (*P *< 0.05) difference between cell types and marker expression. BM-MSC: Bone marrow-derived mesenchymal stem cells; AT-MSC: adipose tissue-derived mesenchymal stem cells.

Isolation of bovine fetal BM-MSC and AT-MSC was performed based on the capacity for plastic attachment under standard culture conditions that included DMEM media supplemented with 10% FBS. Colonies of fibroblast-like cells attached to the plastic were visualized at Days 5–6 after seeding. BM-MSC exhibited characteristic spindle shape and fibroblast morphology, whereas AT-MSC displayed subpopulations of polygonal and fibroblast morphology. Mesenchymal CD73, hematopoietic CD34, pluripotent Nanog, and Oct4 markers expressions were determined in BM-MSC and AT-MSC by FACS analysis. A greater (*P* < 0.001) proportion of BM-MSC and AT-MSC were positive for CD73 (82.6 ± 6.5% and 79.1 ± 3.8%), Nanog (80.6 ± 4.7% and 90.3 ± 3.1%), and Oct4 (93.4 ± 5.2% and 91.1 ± 3.9%) (Figure 1). Moreover, a lower (*P *< 0.001) proportion of BM-MSC were positive for CD34 (7.6 ± 1.2%) compared to AT-MSC (9.7 ± 2.1%).

**Figure 2:**
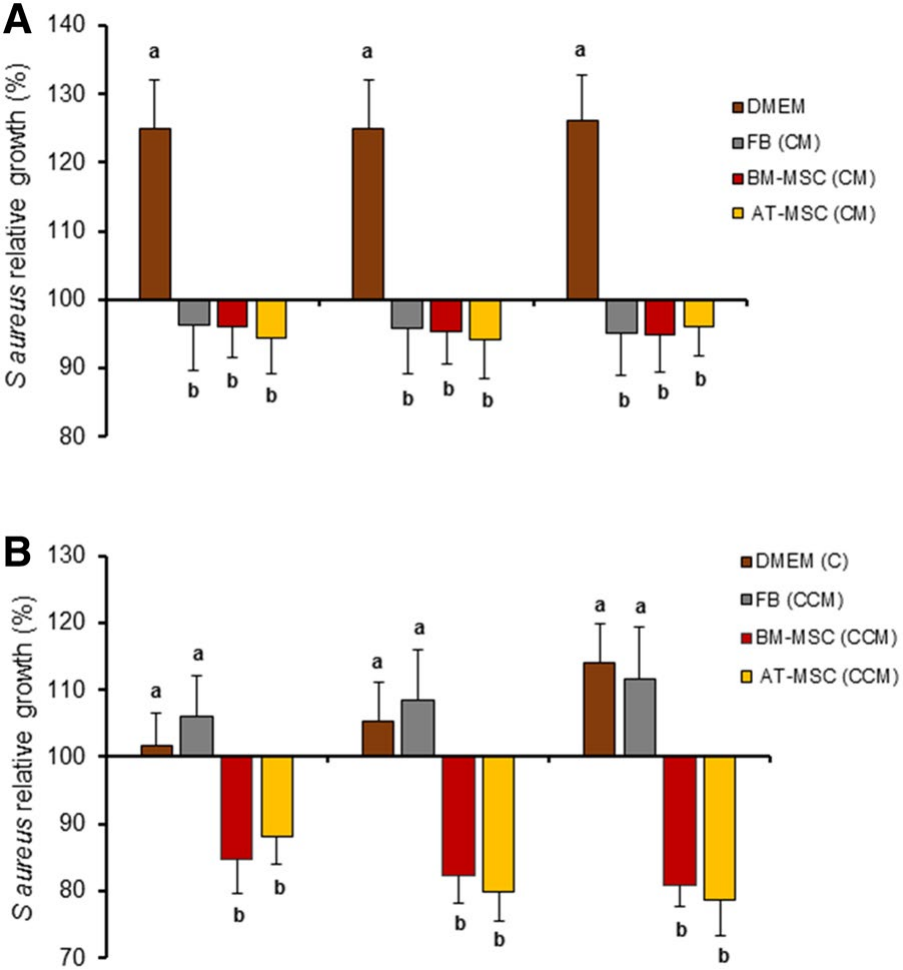
**Relative growth of** ***S. aureus*** **after 1, 2 and 3** **h of incubation in CM and CCM from bovine fetal FB, BM-MSC and AT-MSC. A** *S. aureus* relative growth was lower (*P* < 0.05) in CM from FB, BM-MSC, and AT-MSC compared to relative growth in DMEM (positive control). **B** *S. aureus* relative growth after 1, 2 and 3 h of culture was lower (*P* < 0.05) in CCM from BM-MSC and AT-MSC compared to growth in concentrated DMEM and CCM from FB, respectively. (a, b) Indicate significant (*P *< 0.05) difference between cell type and DMEM. DMEM: Dulbecco’s Modified Eagle Medium; FB: fibroblasts, BM-MSC: bone marrow-derived mesenchymal stem cells; AT-MSC: adipose tissue-derived mesenchymal stem cells; CM: conditioned media; CCM: concentrated conditioned media.

The antibacterial capacity of CM from fetal bovine BM-MSC and AT-MSC was evaluated under in vitro conditions against a strain SAU-1 of *S. aureus* isolated from a clinical mastitis case. Plain medium (DMEM) was included as a positive control and skin fetal FB were also tested considering its reported potential to secrete AP. After 1, 2 and 3 h of culture, *S. aureus* relative growth was lower (*P *< 0.05) in CM from FB (96.4, 95.8, and 95.1%), BM-MSC (96.1, 95.4, and 94.9%) and AT-MSC (94.4, 94.2, and 96.1%) respectively, compared to relative growth in DMEM (124.9, 125, and 126.2%; Figure 2A). Moreover, *S. aureus* relative growth after 1, 2 and 3 h of culture was lower (*P *< 0.05) in CCM from BM-MSC (84.9, 82.3, and 80.9%) and AT-MSC (88.2, 79.8, and 78.8%) compared to relative growth in concentrated DMEM (101.7, 105.4, and 114%) and CCM from FB (106.1, 108.6, and 111.5%), respectively (Figure 2B).

### *Staphylococcus aureus* in vitro relative growth is reduced in presence of activated CM and activated concentrated CM from BM-MSC and AT-MSC

**Figure 3:**
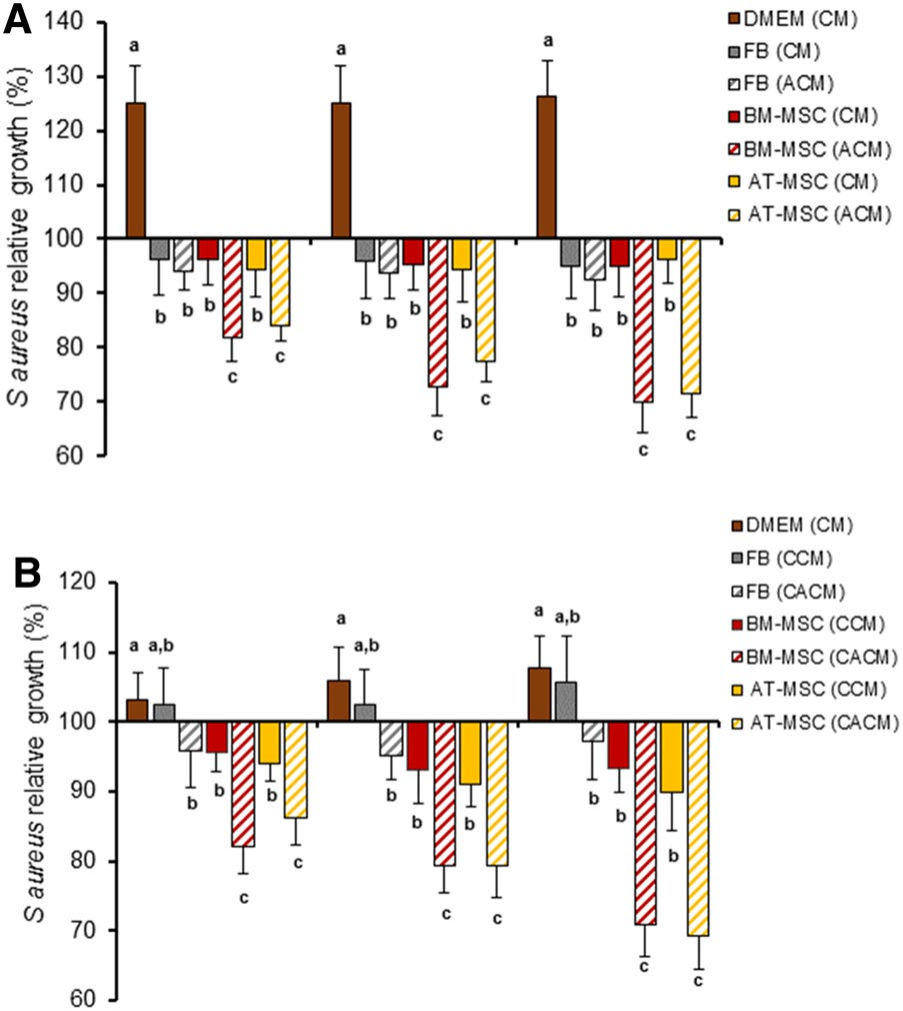
**Relative growth of** ***S. aureus*** **after 1, 2 and 3** **h of incubation in CM, ACM and CACM from bovine fetal FB, BM-MSC and AT-MSC. A** *S. aureus* relative growth was lower (*P *< 0.05) in ACM from BM-MSC and AT-MSC compared to *S. aureus* growth in DMEM, CM from FB, ACM from FB, CM from BM-MSC and CM from AT-MSC. **B** *S. aureus* relative growth was reduced (*P *< 0.05) in CACM from BM-MSC and AT-MSC compared to growth in DMEM, CCM from FB, CACM from FB, and CCM from BM-MSC, CCM from AT-MSC. (a–c) Indicate significant (*P *< 0.05) difference between cell type and DMEM. DMEM: Dulbecco’s Modified Eagle Medium; FB: fibroblasts, BM-MSC: bone marrow-derived mesenchymal stem cells; AT-MSC: adipose tissue-derived mesenchymal stem cells; CM: conditioned media; CCM: concentrated conditioned media; ACM: activated conditioned media; CACM: concentrated activated conditioned media.

The direct contact *of S. aureus* and BM-MSC and AT-MSC was also evaluated on the antibacterial capacity of ACM from BM-MSC and AT-MSC. After 1, 2 and 3 h of culture, *S. aureus* relative growth was lower (*P *< 0.05) in ACM from BM-MSC (81.9, 72.9, and 69.9%) and AT-MSC (83.9, 77.5, and 71.6%) compared to *S. aureus* relative growth in DMEM (124.9, 124.9, and 126.2%), CM from FB (96.4, 95.8, and 95.1%), ACM from FB (94.1, 93.6, and 92.6%), CM from BM-MSC (96.1, 95.4, and 94.9%) and CM from AT-MSC (94.4, 94.2, and 96.1%), respectively (Figure 3A). Similarly, *S. aureus* relative growth after 1, 2 and 3 h of culture was reduced (*P *< 0.05) in CACM from BM-MSC (82, 79.3, and 70.9%) and AT-MSC (86.2, 79.3, and 69.3%) compared to bacterial growth in DMEM (103.1, 106, and 107.7%), CCM from FB (102.4, 102.4, and 105.7%), CACM from FB (95.7, 95.1, and 97.3%), CCM from BM-MSC (95.7, 93.1, and 93.2%), CCM from AT-MSC (94.1, 91, and 89.9%), respectively (Figure 3B).

### Direct contact with *S. aureus* increases bBD4A and NK1 expression in BM-MSC, AT-MSC and FB from bovine fetuses

**Figure 4:**
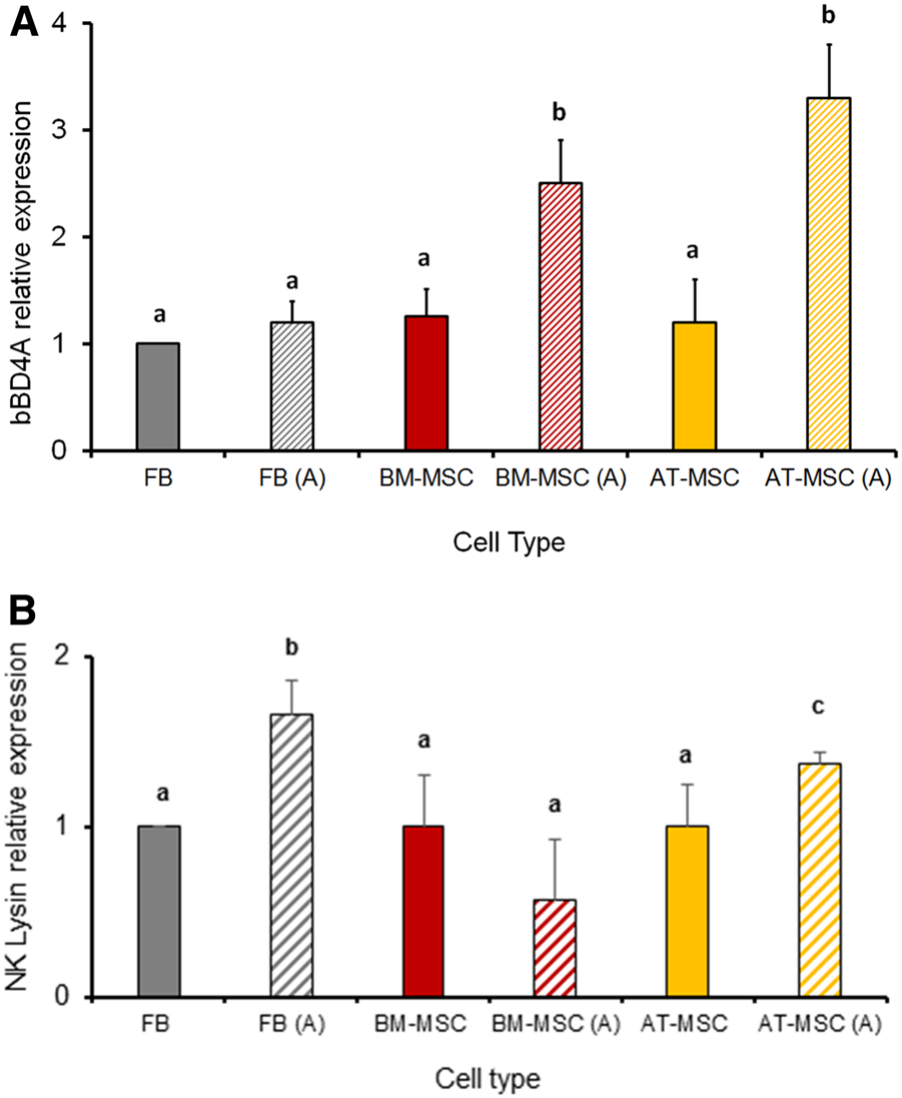
**Analysis of antibacterial related molecules mRNA levels in bovine fetal FB, BM-MSC and AT-MSC activated by exposure to** ***S. aureus*** **for 6** **h. A** bBD4A mRNA levels increased (*P *< 0.05) in activated BM-MSC and AT-MSC compared to non-activated controls. **B** NK1 mRNA levels increased (*P *< 0.05) in activated FB and AT-MSC compared to non-activated controls. (a–c) Indicate significant (*P *< 0.05) difference between activated and no-activated cell type. FB: Fibroblasts, BM-MSC: bone marrow-derived mesenchymal stem cells; AT-MSC: adipose tissue-derived mesenchymal stem cells; A: activated.

**Figure 5:**
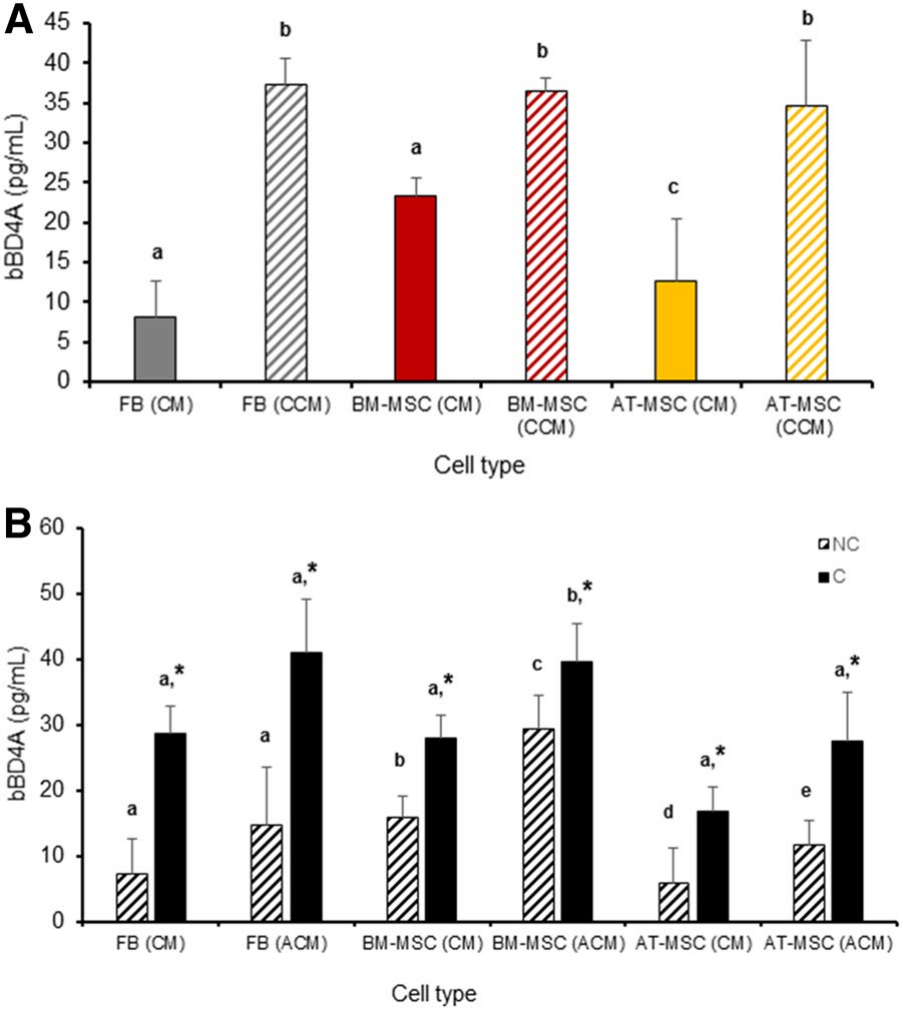
**Analysis of bBD4A protein levels in CM, ACM and CACM from bovine fetal FB, BM-MSC and AT-MSC bovine fetal FB, BM-MSC and AT-MSC activated by exposure to** ***S. aureus*** **for 6** **h.** Higher levels of bBD4A were detected in CCM from FB, BM-MSC and AT-MSC compared to CM. Activation of BM-MSC and AT-MSC increased (*P* > 0.05) bBD4A levels in ACM compared to CM. Moreover, CACM had higher (*P *< 0.05) bBD4A levels in BM-MSC and AT-MSC compared to ACM. (a–d) Indicate significant (*P *< 0.05) difference between CM and CCM (**A**) and CM and ACM (**B**) from each cell type. (*) Indicate significant (*P *< 0.05) difference between CM and CCM from each cell treatment. FB: Fibroblasts, BM-MSC: bone marrow-derived mesenchymal stem cells; AT-MSC: adipose tissue-derived mesenchymal stem cells; CM: conditioned media; CCM: concentrated conditioned media; ACM: activated conditioned media; NC: non-concentrated; C: concentrated.

Levels of mRNA of bBD4A, NK1, CATHL2, HEP and IDO were evaluated in fetal bovine BM-MSC, AT-MSC and FB cultures after direct contact with *S. aureus* (activated MSC). Moreover, concentrations of bBD4A were quantified in CM, ACM and CACM from fetal bovine BM-MSC, AT-MSC and FB. Levels of mRNA of CATHL2, HAMP and IDO were not detected in non-activated or activated MSC. bBD4A mRNA levels increased (*P *< 0.05) 1.2- and 3.5-fold in activated BM-MSC and AT-MSC compared to non-activated controls (Figure 4A). No significant differences in bBD4A mRNA levels were detected between activated and non-activated FB. NK1 mRNA levels increased (*P *< 0.05) 1.4- and 1.7-fold in activated FB and AT-MSC compared to non-activated controls (Figure 4B). Filtration increased bBD4A concentration in CCM from FB, BM-MSC and AT-MSC (37.3, 36.4 and 34.5 pg/mL) compared to CM (8.1, 23.4, 12.7 pg/mL, respectively; Figure 5A). Activation of BM-MSC and AT-MSC increased (*P* > 0.05) bBD4A levels in ACM compared to CM (29.4 vs. 16 and 11.7 vs. 5.8 pg/mL, respectively; Figure 5B). Moreover, CACM had higher (*P *< 0.05) bBD4A levels in BM-MSC and AT-MSC (39.6 and 27.6 pg/mL) compared to ACM (28.1 and 19.9 pg/mL).

## Discussion

To our knowledge, this study is the first reported comparative analyses of bovine fetal BM-MSC and AT-MSC based on antibacterial potentials against mastitis-causing pathogen *S. aureus*. Moreover, this study describes an association between the expression of AP genes bBD4A and NK1 and bBD4A protein and the antibacterial capacity of BM-MSC and AT-MSC. The majority of plastic-adherent populations of BM-MSC and AT-MSC expressed mesenchymal marker CD73 and were negative for hematopoietic marker CD34, indicating that the isolated cell populations exhibited specific profile of MSC [3]. Although, bovine fetal BM-MSC and AT-MSC displayed similar patterns of expression of CD73 and CD34, previous studies have reported lower expression of CD34 in canine, equine and human MSC [18–20]. In this regard, expression of CD34 may be associated to the extension of culture period since freshly isolated MSC express low levels of CD34 but extended cultured MSC lack expression of this protein [21]. Moreover, BM-MSC and AT-MSC expressed similar levels of pluripotent transcriptional factors Nanog and Oct4, suggesting features of multipotent cells.

Despite the antibacterial mechanism of MSC during bacterial exposure has not been completely elucidated, it has been reported that MSC alter their secreted products, migration, proliferation and differentiation upon encounter with bacteria [22]. In the case of *S. aureus*, exposure of human MSC to this pathogen increases production of several paracrine factors including VEGF, SDF-1, and IL-6, that are involved in the influx and activation of inflammatory cells to infected tissue [23]. In vivo experiments indicate that treatment with MSC reduce bacterial infection and inflammatory response in rats infected with methicillin-resistant *S. aureus* [24]. Bacterial clearance in vivo may be associated in part to phagocytosis since sepsis-induced mice treated with MSC reduce inflammation (IL-10 and IL-6) while enhancing phagocytosis of *S. aureus* [25].

Despite reports on immunomodulatory and phagocytic activity of MSC, the antibacterial response against *S. aureus* may be primarily driven by production of AP. These peptides are part of the innate immunity that provides the first line of defense against pathogenic microorganisms [26]. In the present study, the expression of five AP was evaluated, and the expression of bBD4A and NK1 was present and associated to the in vitro antibacterial effect of MSC. bBD4A expression in BM-MSC and AT-MSC, and the antibacterial capacity of their CM was increase after exposure for 6 h with *S. aureus*. In contrast, FB used as negative controls but did not showed upregulation in BD4A mRNA levels or protein expression after exposure to *S. aureus*. In addition, CM from FB did not reduce *S. aureus* proliferation, which suggest that BD4A may be the main AP responsible for the inhibitory effect of CM from BM-MSC and AT-MSC against this pathogen.

In contrast to bBD4A and NK1, the expression of two previously reported MSC-derived AP, CATHL2 and HEP was not detected in BM-MSC or AT-MSC. These results provide evidence on the potential role of selected AP including bBD4A and NK1in the reduction of *S. aureus* growth under in vitro conditions. Defensins constitute a large family of small, cysteine rich, cationic peptides that have been found in various tissues in many animal species and are capable of killing a broad spectrum of pathogens [27]. The lingual antimicrobial peptide (LAP), one of the first characterized bovine β-Defensins, was isolated from swollen tongue of cattle [28]. It has been reported that β-Defensins are present in the cow milk [29] and exhibit antibacterial capacity against *S. aureus* [30]. A positive relationship has been found between somatic cell count (SCC) in milk and LAP mRNA expression, which was localized in epithelial cells of cow mastitic tissue [31]. Moreover, LAP concentration and SCC in milk are also increased after intramammary administration of lipopolysaccharide (LPS) in cows or heat-killed *S. aureus* in goats [32–34]. Overall these results indicate that secretion of β-Defensins is stimulated by *S. aureus* not only in epithelial cells of the mammary gland but also in BM-MSC and AT-MSC derived from bovine fetuses.

Exposure to *S. aureus* augmented mRNA levels of NK1 in AT-MSC but not in BM-MSC. These results suggest that antibacterial strategy against *S. aureus* variates depending on MSC tissue sources. NK1 is a cationic AP firstly isolated from porcine small intestine that is produced by cytotoxic T and NK cells [35]. In the bovine, four NK-lysin genes have been described (NK1, NK2A, NK2B and NK2C) with higher expression in the Peyer´s patch (NK1, NK2A and NK2B) and the lung (NK2C) [36]. All four synthetic forms of NK-lysin peptides had antibacterial capacity against *S. aureus* [36]. Recently, genetics variants within the bovine NK-lysin gene have been reported which potentially provides two candidate genetic markers for association with health-related phenotypes [36].

Indolamine 2,3-dioxygenase, CATHL2 and HEP mRNA levels were not detected in the experiments using bovine fetal MSC. Previous studies have indicated that human MSC require the effect of inflammatory signals such as IFNγ and TNFα, in order to activate expression of IDO [6]. This study also reported antibacterial activity of IDO against of *S aureus*. Conversely, mice MSC lacking expression of IDO do not inhibit *S. aureus* growth [6]. Similarly, in previous in vitro experiments we found that IFNγ treatment induced activation of IDO mRNA expression in bovine fetal MSC and that IDO mRNA levels have a dose–response effect with IFNγ concentration (unpublished data). These studies indicate that exposure to an inflammatory environment is required for MSC in order to express IDO and that this factor is not the exclusive antibacterial factor against *S. aureus*. CATHL2 is another AP that is produced by human and equine MSC and has been involved in the antibacterial capacity of MSC against *S. aureus* [37, 38]. CATHL2 is synthesized by bovine mammary epithelial cells and is up-regulated in response to *S. aureus* [39]. Similarly, HAMP activity has also been associated to *S. aureus* inhibition [40] and its expression has been reported in menstrual-derived MSC [7]. The lack of expression of CATHL2 and HEP in bovine fetal MSC after exposure to *S. aureus* may be associated to a tissue-specific localization and regulation of these peptides; however, further analyses may be required in order to determine the potential participation of these factors after exposure to different pathogens.

Our data indicate that CM from fetal bovine BM-MSC and AT-MSC has the capacity to reduce in average ~30% of *S. aureus* relative growth under in vitro conditions. Moreover, concentration by filtration and pre-exposure to *S. aureus* increased antibacterial capacity of CM against *S. aureus*. From four AP evaluated, expression of two of them, bBD4A and NK1 was present and associated to the in vitro antibacterial effect of MSC.

## Abbreviations

BM-MSC: bone marrow-derived mesenchymal stem cells
AT-MSC: adipose tissue-derived mesenchymal stem cells
FB: fibroblasts
CM: conditioned media
CCM: concentrated conditioned media
ACM: activated conditioned media
CACM: concentrated activated conditioned media
DMEM: Dulbecco’s Modified Eagle medium
FBS: fetal bovine serum
PBS: phosphate-buffered saline
AP: antibacterial peptides
bBD-4A: β-defensin 4A
NK1: NK-lysine 1
CATHL2: cathelicidin 2
HEP: hepcidin
IDO: indoleamine 2,3 dioxygenase
FBS: fetal bovine serum
FACS: fluorescence-activated cell sorter
Q-PCR: quantitative-polymerase chain reaction
